# Spatial Proteomics Reveals Alcohol-Induced Damages to the Crypts and Villi of the Mouse Small Intestine

**DOI:** 10.1021/acs.jproteome.4c00037

**Published:** 2024-04-24

**Authors:** Patil Shivprasad Suresh, Xinguo Sun, Zhanxiang Zhou, Qibin Zhang

**Affiliations:** † Center for Translational Biomedical Research, University of North Carolina at Greensboro, North Carolina Research Campus, Kannapolis, North Carolina 28081, United States; ‡ Department of Chemistry & Biochemistry, University of North Carolina at Greensboro, Greensboro, North Carolina 27402, United States; § Department of Nutrition, University of North Carolina at Greensboro, Greensboro, North Carolina 27402, United States

**Keywords:** Paneth cell, crypts, villi, alcohol, DIA proteomics, laser capture microdissection

## Abstract

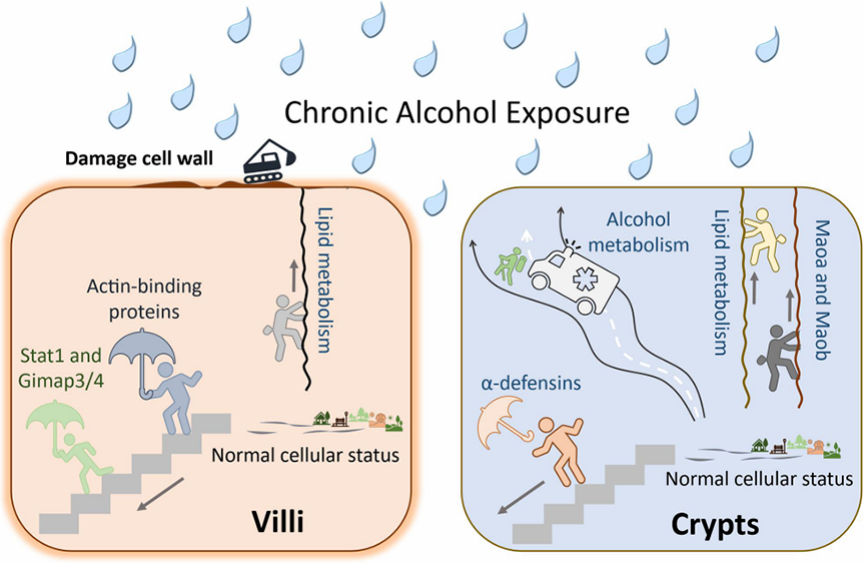

Alcohol consumption perturbs the gut immune barrier and ultimately results in alcoholic liver diseases, but little is known about how immune-related cells in the gut are perturbed in this process. In this study, we employed laser capture microdissection and a label-free proteomics approach to investigate the consequences of alcohol exposure to the proteomes of crypts and villi in the proximal small intestine. Intestinal tissues from alcohol-fed and pair-fed mice were microdissected to selectively capture cells in the crypts and villi regions, followed by one-pot protein digestion and data-independent LC–MS/MS analysis. We successfully identified over 3000 proteins from each of the crypt or villi regions equivalent to ∼3000 cells. Analysis of alcohol-treated tissues indicated an enhanced alcohol metabolism and reduced levels of α-defensins in crypts, alongside increased lipid metabolism and apoptosis in villi. Immunofluorescence imaging further corroborated the proteomic findings. Our work provides a detailed profiling of the proteomic changes in the compartments of the mouse small intestine and aids in molecular-level understanding of alcohol-induced tissue damage.

## Introduction

The crypts of Lieberkühn in the small intestine are composed of a heterogeneous consortium of functionally diverse cell types, including Paneth cells and stem cells. Paneth cells are defensive epithelial cells that secrete antimicrobial peptides to protect the gut against enteric infections and maintain the gut microbial composition and translocation.^1^ Additionally, they play pivotal roles in regulating the proliferation and differentiation of the intestinal epithelium.^2^ Paneth cells secrete antimicrobial peptides like α-defensin, lysozyme, and phospholipase A, along with inflammatory cytokines that can be promptly triggered and released when exposed to diverse microbial or inflammatory stimuli.^3^ The villi are composed primarily of enterocytes for absorption of nutrients, and the remaining Goblet and Tuft cells are sparsely distributed for secretion of mucins and for chemosensing.^4^ Excessive alcohol consumption leads to enhanced penetrability in the intestinal barriers, enabling translocation of pathogen-associated molecular patterns to the liver and ultimately resulting in steatohepatitis,^5,6^ but the intracellular consequences of these cells and the status of antimicrobial peptides during exposure to alcohol remain poorly understood.

A thorough exploration of alcohol’s effects on crypts and villi, with a particular focus on Paneth cell functionality, is imperative. However, analyzing the proteome of the entire proximal small intestinal tissue fails to offer cell-specific insights, and culturing Paneth cells proves to be challenging because of their dependence on coexisting epithelial and stem cells for viability. Laser capture microdissection (LCM) resolves this issue by microscopically isolating the desired population of cells from a frozen or fixed tissue section;^7^ when in combination with mass spectrometry-based proteomics, it enabled the application of spatial proteomics to improve understanding of biological processes and disease etiology.^8−11^

We recently developed a workflow comprising a simple tissue staining procedure, LCM, one-pot protein digestion and disposable trap-based liquid chromatography with tandem mass spectrometry (LC–MS/MS) analysis to comprehensively profile the Paneth cell proteome in mouse intestine tissue.^12^ Here, based on that workflow and using a more sensitive data-independent acquisition (DIA)-based proteomics approach, we explored the molecular impacts of alcohol on the small intestine’s crypts and villi regions. This represents the first spatial proteomics attempt to decipher the molecular events at the proteome scale that occur in the crypts and villi under alcohol treatment. Interesting damaging pathways were uncovered as responses to alcohol exposure to the crypt and villi cell populations, and the expression patterns of critical proteins were verified through immunofluorescence imaging.

## Experimental Procedures

### Alcohol Feeding and Preparation of Cryostat Intestinal Tissue Sections

C57BL/J mice at 12-week-old were fed an alcohol-containing Lieber-DeCarli liquid diet (alcohol-fed; AF, *n* = 9) or an isocaloric control liquid diet (pair-fed; PF, *n* = 7) for 8 weeks. The Lieber-DeCarli regular alcohol (DYET #710260) and control (DYET #710027) liquid diets for rodents were purchased from the Dyets Inc. (Bethlehem, PA), and liquid diet was prepared according to the following instructions: for alcohol diet, add 67 mL of 95% ethanol to 132.18 g of DYET #710260 and mix with cold water to 1 L; for isocaloric control diet, mix 221.78 g of DYET #710027 with cold water to 1 L. The AF mice were fed ad libitum, whereas the PF mice were fed with the same amount of liquid diet consumed by the AF group in the previous day. At the end of alcohol feeding, intestinal tissue samples were taken from the ileum, placed in a Tissue-Tek optimal cutting temperature (OCT) compound, snap frozen in liquid nitrogen, and stored at −80 °C. Cryostat sections of the OCT-embedded intestinal tissues were cut at 10 μm for LCM and 5 μm for immunofluorescence. All animal work was performed under protocol 21-014 and approved by the IACUC of the North Carolina Research Campus.

### Tissue Staining

The mouse intestinal tissue sections, affixed to PEN membrane slides, were allowed to reach room temperature after resting for 5 min. Subsequently, these slides were gently immersed in 1X phosphate-buffered saline solution (Thermo Scientific, catalog number 70011-044) for a 5 min wash, facilitating the removal of the OCT compound. The slides were then carefully removed to eliminate excess solution and dropwise covered the entire tissue section with a 0.5% w/v solution of toluidine blue O (TBO; Sigma-Aldrich, catalog no. 1159300025) for 1.5 min. Then, the slides underwent sequential rinsing in two separate containers, first for 20 s and then for 1 min, using Milli-Q water. The thoroughly cleansed tissue sections were positioned face up on a paper towel, allowing them to air-dry for 5 min before microdissection procedures.

### Laser Capture Microdissection

The TBO-stained tissue slides were meticulously examined, and regions of interest were thoughtfully selected for precise dissection according to the tissue morphology. This dissection process was performed using a ZEISS PALM microbeam microscope (ZEISS, Oberkochen, Germany) controlled by PALMRobo software (version 4.6). The microbeam was configured with an energy setting of 38 and a cutting speed of 12. Subsequently, the dissected tissues were captured into 0.2 mL PCR tube caps, which had been preloaded with 20 μL of 50 mM TEABC (triethylammonium bicarbonate buffer; Sigma-Aldrich, catalog number T7408). The crypts (containing Paneth and stem cells) and the villi (containing columnar and goblet cells) regions were dissected from a total of nine AF and seven PF mouse intestinal samples, each with a population of approximately 3,000 cells. Once the specimens were collected, the tubes were capped and centrifuged at 5000*g* for 10 min to spin down the tissue pieces for in-solution digestion.

### In-Solution Digestion

The crypts and villi tissue samples were digested in the same tube containing 20 μL of 50 mM TEABC, with the addition of 0.2% DDM (*n*-dodecyl-β-d-maltoside; Sigma-Aldrich, catalog number D4641) in 50 mM TEABC, followed by sonication at 37 °C for 10 min. The resulting lysate was reduced by adding 5 mM DTT (dithiothreitol) and subsequent incubation at 37 °C for 60 min, followed by alkylation using 12.5 mM IAA (iodoacetamide; Sigma-Aldrich, catalog number I6125) at 37 °C for 60 min in the dark. To facilitate proteolytic digestion, 0.5 μg of trypsin/LysC (protease mix; Thermo Scientific, catalog number A40009) was introduced, and the mixture was allowed to incubate overnight at 37 °C. Finally, the digested peptides were acidified with 1% formic acid, effectively terminating the enzymatic digestion process.

### LC–MS/MS Data Acquisition

Following the manufacturer’s guidelines, the digested peptide samples were loaded onto EvoTip trap columns (Evosep, catalog number EV2013). This process involved washing the EvoTips with 20 μL of solvent B (acetonitrile with 0.1% formic acid), conditioning with 100 μL of 2-propanol, and equilibrating with 60 μL of Solvent A (water with 0.1% formic acid). Post-sample loading, the tips underwent two 60 μL washes with Solvent A, followed by a final wash with 100 μL of Solvent A to prevent the tips from drying. All the Evosep sample loading steps were carried out in a centrifuge for 60 s at 800 g. Subsequently, peptides on the EvoTips were separated using a 15 cm × 150 μm EASY-Spray column (PepMap RSLC C18, catalog number ES906, packed with 2 μm C18 beads) on an Evosep One LC system (Evosep, Denmark). Peptides were eluted with solvent B concentration of less than 35% at a flow rate of 0.5 μL/min using the 44 min gradient, 30 samples per day method. Peptides were detected in positive ion mode by utilizing an Orbitrap Exploris 240 mass spectrometer (OE240; Thermo Fisher). Mass spectra were acquired within the range of 390 to 1010 *m*/*z* at a mass resolution of 60 k (at 200 *m*/*z*), followed by DIA MS/MS with a mass isolation window of 24 *m*/*z* and a mass resolution of 30 k (at 200 *m*/*z* 200). Other critical settings on the OE240 included a normalized HCD collision energy of 30, normalized AGC target: 300% for MS1 scan and 1000% for the DIA scan, and ion injection time: 100 ms for the MS1 scan and 55 ms for the DIA scan.

### Proteomics Data Processing

The DIA raw data from the experiment were analyzed using DIA-NN (version 1.8.1; https://github.com/vdemichev/DiaNN).^13^ The *Mus musculus* database was employed for protein identification, with reannotation enabled, and the database was downloaded from Uniprot on June 15, 2023. The analysis incorporated various settings, including the FASTA digest for library-free search/library generation and the utilization of deep learning-based algorithms for spectra and retention time (RTs) prediction. Critical parameters such as mass accuracy, MS1 accuracy, and scan window were configured to 15.0, 20.0, and 4, respectively. Enzyme specificity was defined as trypsin with an allowance for one missed cleavage. Carbamidomethyl modification on cysteine residues was set as a fixed modification, and a match between runs was enabled to enhance data alignment. Protein inference was grouped on genes, employing a neural network classifier in single-pass mode, and quantification was optimized for LC with a high accuracy. Further, cross-run normalization was tailored to RT-dependent dynamics, while library profiling employed smart profiling techniques. The remaining parameters were kept at their default settings for a comprehensive analysis.

### Statistics Analysis

The output files generated by DIA-NN underwent statistical analysis employing Perseus software (version 1.6.14.0; https://maxquant.net/perseus/).^14^ This entailed a logarithmic transformation (log_2_) and normalization using the width adjustment normalization algorithm. Significantly altered proteins were determined through a student’s *t* test, with a permutation-based false discovery rate (FDR) set at <0.05 and an *S*_0_ value at <0.05. Further data exploration involved a volcano plot, heat map, and principal component analysis (PCA) analysis, all executed within the Perseus framework.

### Bioinformatics Analysis

The list of significantly altered proteins within the enriched cell population underwent comprehensive functional enrichment analysis using STRING (version 12.0; https://string-db.org/).^15^ The most prominent biological processes enriched in the cell populations were chosen to epitomize their functional roles. To further refine our understanding of these processes, the STRING database generated protein–protein interaction networks and subsequently fine-tuned through the utilization of Cytoscape (Version 3.10.0).^16^ Moreover, we employed the Metascape bioinformatics tool (Version 3.5, last updated on 05/01/2023; https://metascape.org/)^17^ to scrutinize and elucidate the common or distinctive enriched functions within the clusters of enriched proteins, providing a comprehensive insight into their functional significance.

### Immunofluorescence

The immunofluorescence procedure was conducted on intestinal tissue sections, cryosectioned from the same AF and PF intestine samples used for LCM work. These tissue sections, mounted on standard glass slides, were initially subjected to a 30 min block with normal donkey serum at room temperature. Subsequently, the cryostat sections were incubated with rabbit anti-Adh1 (Abcam, Waltham, MA), rabbit anti-Aldh1b1 (Santa Cruz Technology, Dallas, TX), rabbit anti-Casp8 (Cell signaling Technology, Danvers, MA), or anti-Stat1 (Cell signaling Technology, Danvers, MA) overnight at 4 °C. Following this, Alexa Fluor 594-conjugated donkey antirabbit IgG (Jackson ImmunoResearch Laboratories, West Grove, PA) was applied to the tissue sections for a 30 min incubation at room temperature. To visualize the nuclei, tissue slides were counterstained with 4′,6-diamidino-2-phenylindole (DAPI) (Thermo Fisher Scientific). Fluorescence intensity was quantified using an Image-Pro V10 software (Media Cybernetics, MD) and statistical differences between the PF and AF groups were analyzed by *t* test (*n* = 14 from 3 mice in each group).

## Results

**Figure 1:**
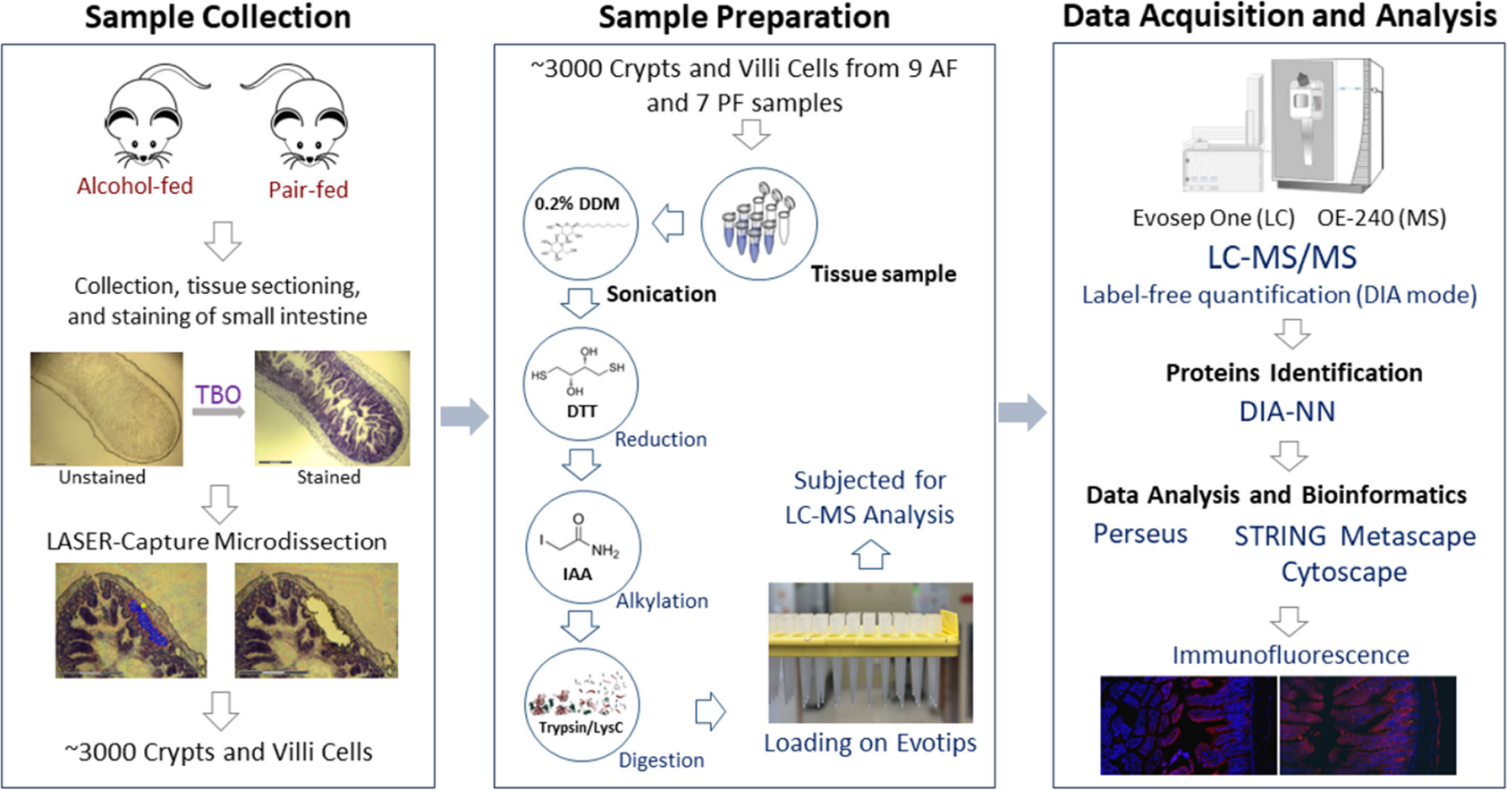
Schematic illustration of the workflow for the LCM-based proteomics study. The cells of crypts and villi regions (equivalent to ∼3000 cells) were dissected from alcohol-fed and pair-fed mouse small intestine samples using LCM. Each scale bar within the image corresponds to 300 μm. The collected tissue samples were subsequently subjected to in-solution digestion and loading onto Evotips for further analysis by using LC–MS/MS. The output raw files underwent rigorous protein identification and comprehensive statistical and bioinformatic analyses to unravel the underlying molecular mechanisms. The expression patterns of some essential proteins were further validated with immunofluorescence imaging.

To gain insight into the molecular impacts of alcohol consumption on the small intestine, we performed a comprehensive spatial proteomics study wherein LCM was employed to isolate crypts and villi tissues, each equivalent to ∼3000 cells, from mice subjected to AF and PF. The workflow of the LCM-based proteomics study is illustrated in Figure 1. Overall, more than 3000 proteins (with a <1% FDR at both the peptide and protein levels) were identified using a DIA approach from each tissue type under each treatment condition (Supplementary Table S1), which more than doubled the number of proteins identified in comparison to a DDA approach applied in our previous study.^12^ More details are presented in the following sections.

### Effects of Alcohol Treatment on the Crypt Proteome

**Figure 2:**
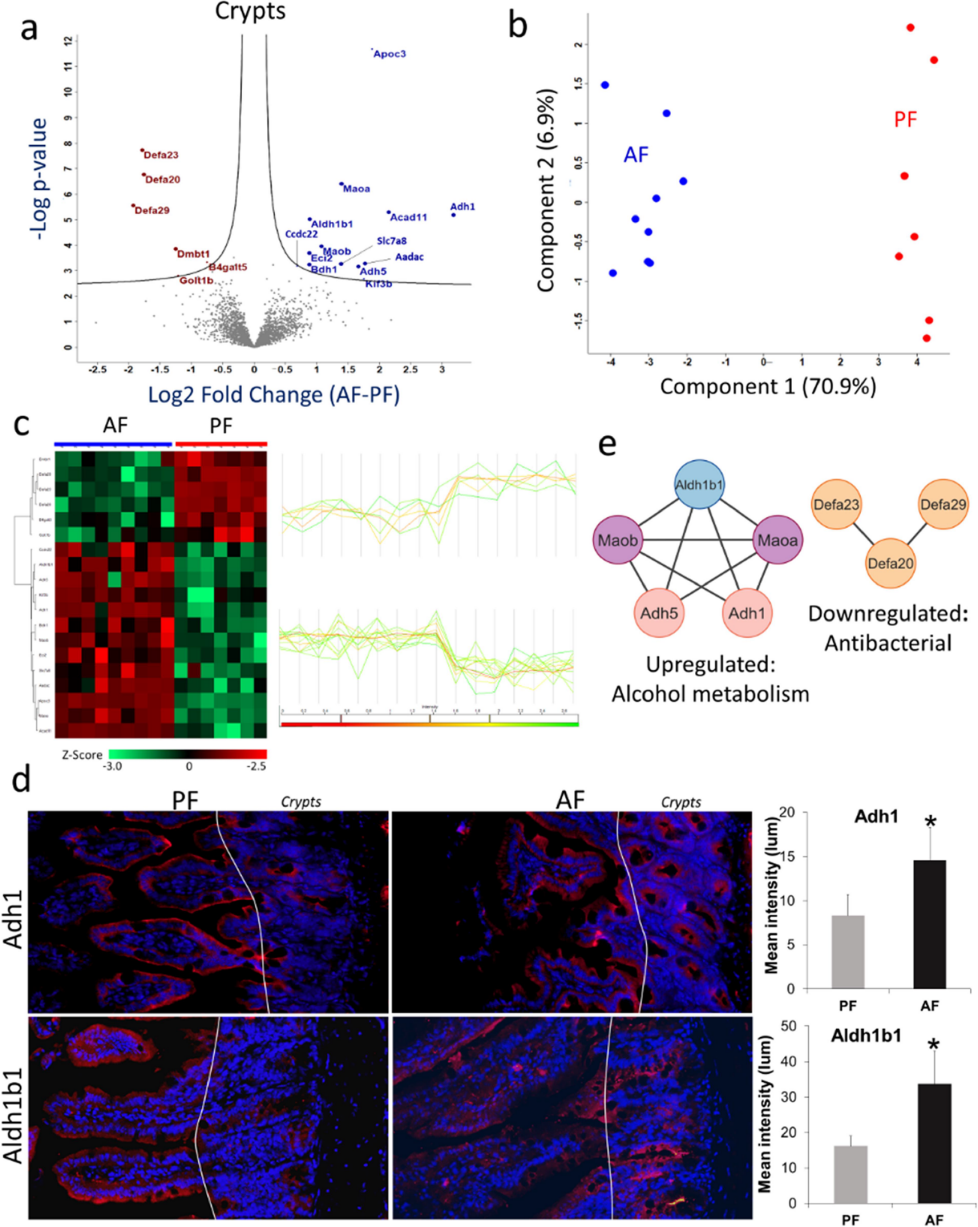
LCM-assisted spatial proteomics profiling of alcohol-treated mouse intestine crypts. (a) Volcano plot between the AF and PF samples of crypts. Student’s *t* test was used with criteria of FDR and *S*_0_ ≤ 0.05. All the enriched proteins are labeled with gene symbols in blue (upregulated) and brown font (downregulated). (b) PCA score plot shows the separation of AF and PF crypt samples with a distinguishing power of 77.8% using the differentially expressed proteins. (c) Heatmap with hierarchical clustering analysis and profile plot of the differentially expressed proteins (Student’s *t* test, FDR < 0.05) in crypts upon alcohol treatment. Protein abundances were log2 transformed before *Z*-score transformation. Profile plots are associated with proteins enriched in each cluster. High signifies increase in relative abundance, and low for decrease in relative abundance. (d) Fluorescent microscopy of crypts (Paneth cells) in the small intestine of AF and PF mice. Immunofluorescence staining validated the expression of Adh1 and Aldh1b1 (upregulated) in the crypts. Red marks and their intensity indicate the targeted proteins’ expression level in the AF and PF, while the blue marks for DAPI counterstained to nuclei. (e) Protein–protein interaction network of up- and down-regulated proteins in crypts.

In total, we identified 3767 and 3764 proteins from the AF and PF crypt regions, respectively (Supporting Information Table S2). Statistical analysis (*t* test with permutation-based FDR correction) identified 19 proteins, 13 upregulated and 6 downregulated proteins upon alcohol treatment (Figure 2a). Unsupervised PCA of these differentially expressed proteins revealed a distinct segregation between the treated (AF) and control (PF) samples, with 77.8% of the variance explained by the first two principal components (Figure 2b). These proteins were also clustered very well in hierarchical clustering based on Pearson's correlations (Figure 2c).

The downregulated proteins are mostly antimicrobial peptides and Paneth cell biomarkers, as exemplified by α-defensin 20 (Defa20), α-defensin 23 (Defa23), and α-defensin 29 (Defa29), with Log_2_ fold-change (FC) values of −1.75, −1.78, and −1.92, respectively (Figure 2a and Supporting Information Table S3). Conversely, enzymes associated with alcohol metabolism exhibited marked upregulation, such as alcohol dehydrogenase 1 (Adh1), alcohol dehydrogenase 5 (Adh5), and aldehyde dehydrogenase 1 family member B1 (Aldh1b1), with Log_2_FC values of 3.18, 1.66, and 0.88, respectively. Furthermore, enzymes involved in lipid metabolism, including apolipoprotein c-III (Apoc3), arylacetamide deacetylase (Aadac), enoyl-CoA delta isomerase 2 (Eci2), and 3-hydroxybutyrate dehydrogenase 1 (Bdh1), also showed an increased abundance in the crypts of AF mice (Figure 2a). To validate the proteomics findings, we checked the expression of Adh1 and Aldh1b1 in AF and PF intestinal tissue sections using immunofluorescence. Compared to background or weak staining of Adh1 and Aldh1b1 in the PF mice, marked increases in the intensity of these two proteins were observed in the crypt area of AF mice (Figure 2d).

To reveal the potential mechanisms underlying alcohol-mediated key alterations in small intestinal cellular processes and signaling pathways, we conducted a gene ontology enrichment analysis using the STRING database. In the context of alcohol-induced changes within the crypts, several significant biological pathways were enriched. Notably, ethanol oxidation, biogenic amine deamination, phase I functionalization of compounds, and metabolic pathways were elevated. Adh1, Adh5, and Aldh1b1 primarily mediate ethanol oxidation; Maoa and Maob oxidatively deaminate amines to aldehydes; and most other upregulated crypt proteins, except for Ccdc22, Kif3b, and Slc7a8, were associated with diverse metabolic pathways. Proteins for phase I functionalization of compounds consist of the alcohol metabolism proteins and Aadac, which mainly involve modifying substrates through enzymatic processes, such as oxidation, reduction, or hydrolysis. Conversely, functions related to antibacterial humoral response and defense against Gram-negative bacteria were suppressed in the crypts (Figure 2e). Notably, Dmbt1 (deleted in malignant brain tumors 1 protein), alongside defensins, plays a pivotal role in antimicrobial and antiviral functions through interacting with innate immunoproteins surfactant A and D.^18^

### Effects of Alcohol Treatment on the Villi Proteome

**Figure 3:**
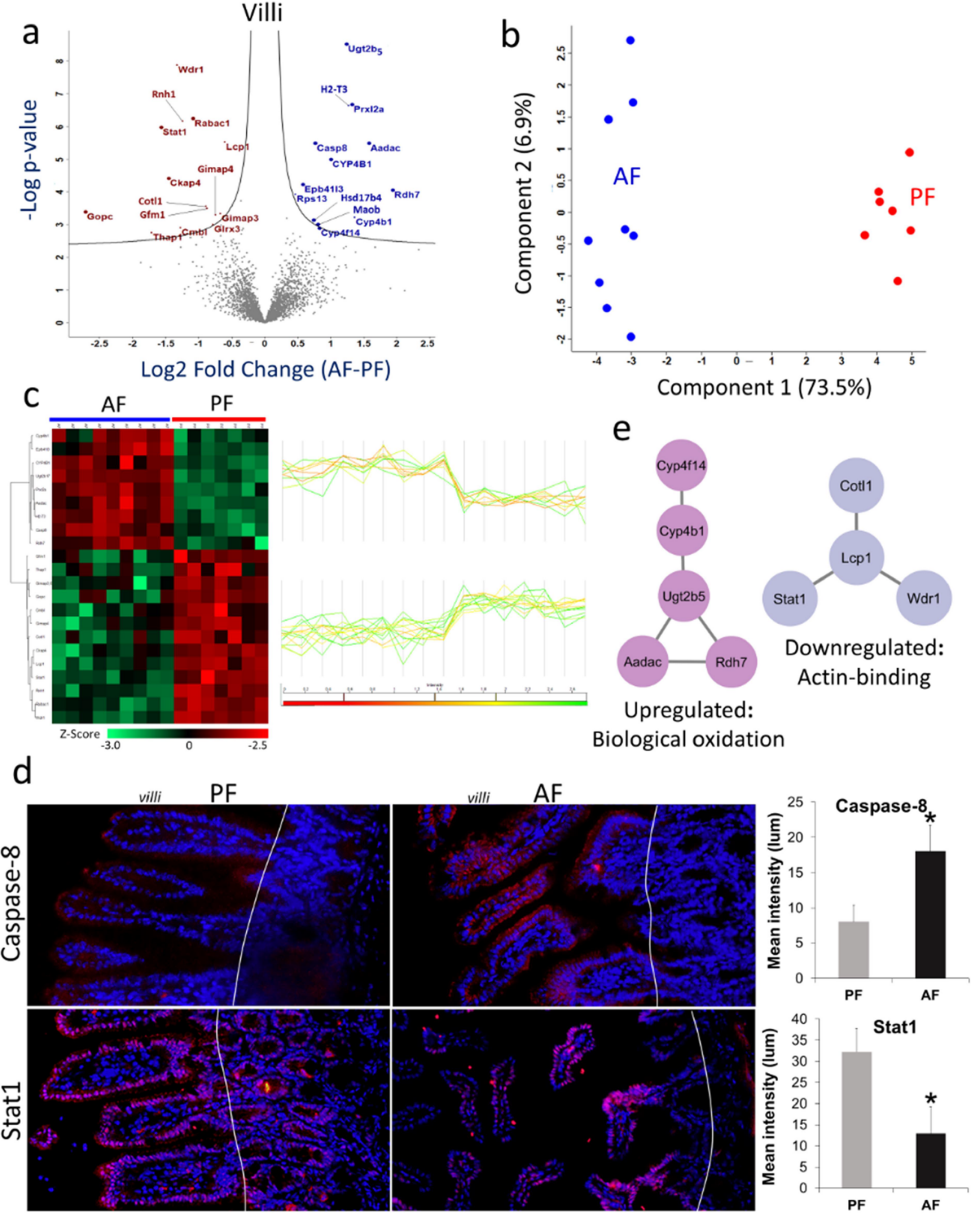
LCM-assisted spatial proteomics profiling of alcohol-treated mouse intestine villi. (a) Volcano plots between the AF and PF samples of villi. Student’s *t* test was used with criteria of FDR and *S*_0_ ≤ 0.05. All the enriched proteins are labeled with gene symbols in blue (upregulated) and brown font (downregulated). (b) PCA score plot shows the separation of AF and PF villi samples with a distinguishing power of 80.4% using the differentially expressed proteins. (c) Heatmap with hierarchical clustering analysis and profile plot of the differentially expressed proteins (Student’s *t* test, FDR < 0.05) in villi upon alcohol treatment. Protein abundances were log2 transformed before *Z*-score transformation. Profile plots are associated with proteins enriched in each cluster. High signifies increase in relative abundance, and low for decrease in relative abundance. (d) Fluorescent microscopy of villi in the small intestine of AF and PF mice. Immunofluorescence staining validated the expression of caspase-8 (upregulated) and Stat1 (downregulated) in villi. Red marks and their intensity indicate the targeted protein expression level in the AF and PF, while the blue marks for DAPI counterstained to the nuclei. (e) Protein–protein interaction network of up- and down-regulated proteins in villi.

In the case of villi, we identified 3981 and 3831 proteins from the AF and PF, respectively (Supporting Information, Table S2). Statistical analysis (*t* test with permutation-based FDR correction) identified 22 proteins, 9 upregulated and 13 downregulated proteins upon alcohol treatment in the villi (Figure 3a). Unsupervised PCA of these differentially expressed proteins revealed a distinct segregation between the AF and the PF, with 80.4% of the variance explained by the first two principal components (Figure 3b). These proteins also clustered very well in hierarchical clustering based on Pearson's correlations (Figure 3c).

Several proteins were suppressed in the villi under alcohol consumption, including signal transducer and activator of transcription 1 (Stat1, Log_2_FC = −1.56), cytoskeleton-associated protein 4 (Ckap4, Log_2_FC = −1.45), glutaredoxin 3 (Glrx3, Log_2_FC = −0.78), GTPase, IMAP family member 4 (Gimap4, Log_2_FC = −0.74), and Gimap3 (Log_2_FC = −0.67) (Figure 3a and Supporting Information Table S3). On the other hand, metabolism-associated proteins were observed as upregulated, such as retinol dehydrogenase 7 (Rdh7, Log_2_FC = 1.74), arylacetamide deacetylase (Aadac, Log_2_FC = 1.57), cytochrome P450 family 4 subfamily B member 1 (Cyp4b1, Log_2_FC = 1.36), leukotriene-B4 omega-hydroxylase 3 (Cyp4f14, Log_2_FC = 0.82), caspase-8 (Casp8, Log_2_FC = 0.76), and hydroxysteroid 17-beta dehydrogenase 4 (Hsd17b4, Log_2_FC = 0.74) (Figure 3a and Supplementary Table S3). To validate the proteomics findings, we assessed the expression of Casp8 and Stat1 in AF and PF intestinal tissue sections using immunofluorescence. In the PF mice, weak cytoplasmic staining of Casp8 was detected in the villi epithelial cells, while moderate to strong nuclear staining of Stat1 was detected in the nuclei of both villi and crypt epithelial cells. Alcohol feeding remarkably increased the intensity of Casp8 but decreased the intensity of Stat1 (Figure 3d).

Using gene ontology enrichment analysis, we further explored the biological pathways associated with altered proteins. Alcohol consumption promotes pathways related to eicosanoid synthesis, including leukotrienes (LT) and eoxins (EX), phase I functionalization of compounds, biological oxidations, and fatty acid metabolism. Cytochromes, specifically Cyp4b1 and Cyp4f14, played critical roles in the synthesis and metabolism of eicosanoids and leukotrienes.^19^ Additionally, these cytochromes and Hsd17b4 were associated with fatty acid metabolism,^20^ while their association with Ugt2b5 and Aadac contributed to biological oxidation (Figure 3e). Conversely, the actin-depolymerizing function in the villi was suppressed by alcohol consumption, which can be attributed to damage to the cytoskeleton system (Figure 3e).

## Discussion

In our previous Paneth cell proteomics study,^12^ using the optimized staining and proteolytic digestion protocol, we were able to identify 1532 proteins from 3600 LCM collected cells from either the crypts or villi regions using the data-dependent acquisition (DDA) MS/MS mode. In contrast, the current study, employing a DIA mode, identified 4230 proteins in AF and 3900 proteins in PF from 3000 cells, representing a more than twofold protein identification rate using the DIA mode compared to the DDA mode. The higher identification rate likely also resulted from using a DIA-NN-based data analysis approach for processing the DIA data, as opposed to the FragPipe-based data processing for the DDA data in our previous study.

**Figure 4:**
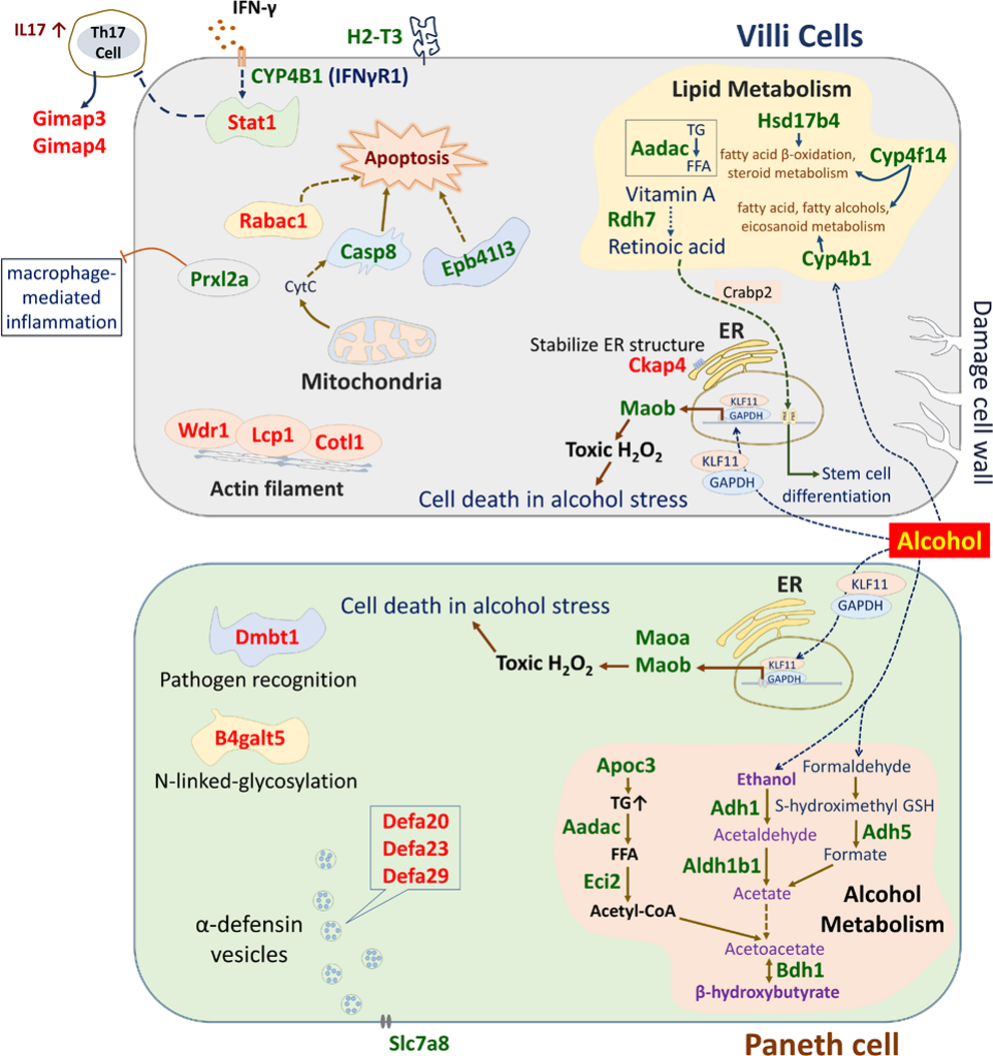
Proposed molecular consequences of alcohol consumption in crypts (paneth cells) and villi. Proteins upregulated: green font; and downregulated: red font.

Upon alcohol treatment, we observed in crypts upregulation of proteins associated with alcohol metabolism, including Adh1, Adh5, and Aldh1b1; lipid metabolism, including Apoc3, Aadac, Bdh1, Acad11, and Eci2; and monoamine metabolism, e.g.*,* Maoa and Maob (Figure 4). In crypts, stem cells coexist with Paneth cells and are vulnerable; increased alcohol metabolism likely reflects increased activities toward detoxification of alcohol and its metabolite aldehyde to maintain a hemostatic environment for stem cell differentiation. In addition, lipid metabolism upregulation has been reported as a consequence of alcohol consumption,^21^ however, ramping up the metabolism of monoamines is novel. On the other hand, downregulation of antimicrobial proteins such as Defa20, Defa23, Defa29, and Dmbt1 was observed in the present study, which agrees well with a previous study that production of α-defensins is notably decreased by Paneth cells in a chronic alcoholic hepatitis mouse model.^6^ The decrease in the levels of α-defensins was linked to changes in the gut microbiota over time, enhanced gut permeability, and the presence of endotoxins.^22^ However, another important antimicrobial enzyme characteristic of Paneth cell–lysozyme did not change upon alcohol treatment, indicating that the Paneth cell population remains stable and the downregulation of defensins is unlikely a result of Paneth cell death. In villi, alcohol also upregulates proteins associated with lipid metabolism. In addition, several other proteins associated with apoptosis (Casp8 and Epb41l3), IFNγ signaling (CYP4B1), H_2_O_2_-mediated cell death (Maob), and retinol metabolism (Rdh7) were upregulated (Figure 4). Notably, the upregulation of Maob and Aadac was observed in both crypts and villi upon alcohol consumption, suggesting alcohol-induced stress can be common in these distinct tissue types within AF. Conversely, downregulated proteins are mostly involved with independent cellular organelle-associated functions, including actin-binding proteins (Colt1, Lcp1, Wdr1), and stabilizing endoplasmic reticulum structure (Ckap4), which may explain the damaged villi structure after prolonged alcohol exposure (Supplementary Figure S1a).

Despite the morphological alterations, especially in the villi induced by alcohol treatment, a shared set of 3615 proteins was identified in the AF and PF (Supplementary Figure S1b). These proteins were associated with key biological processes, including RNA metabolism, signaling by Rho GTPases, vesicle-mediated transport, M phase, ribonucleoprotein complex biogenesis, and neutrophil degranulation (Supporting Information, Figure S2). Besides that, 615 and 285 proteins were uniquely identified in the AF and PF, respectively, indicating that alcohol modulates distinct biological processes in the proximal small intestine. The uniquely expressed AF proteins were associated with mitochondrial translation elongation, mitochondrial translation termination, transcriptional regulation via TP53, cellular response to stress, and regulation of TP53 activity (Supplementary Figure S3). A recent study showed that TP53 plays a crucial role in stimulating the recovery of stem cells within the regenerating intestine following extensive radiation damage.^23^ While exposed to hormonal or chemical triggers, TP53 actively stimulates the differentiation of both mouse and human embryonic stem cells.^24^ In the present work, alcohol may act as a trigger for TP53-mediated functions.

Considering all findings, we postulate the likely molecular events consequential to alcohol consumption in the small intestine (Figure 4). In the crypts (Paneth cells), ethanol and retinol are converted to acetate and retinoic acid, respectively, by Adh1 and Aldh1b1. In parallel, Adh5 is involved in converting methanol to formate, which is subsequently converted into acetate. Upon alcohol treatment, Apoc3 elevates triacylglycerol (TG) level, which is hydrolyzed by Aadac to generate free fatty acid (FFA), and FFA is further converted into acetyl-CoA via β-oxidation process by Eci2. Acetate and acetyl-CoA are further converted into acetoacetate, and Bdh1 converts acetoacetate into β-hydroxybutyrate (BHB), which acts as an endogenous agonist for hydroxycarboxylic acid receptor 2 (HCA2)^25^ and G_i/o_-coupled G protein-coupled receptor (GPCR),^26^ while acting as an antagonist for histone deacetylase^27^ and NLRP3 inflammasome.^28^ Besides, retinoic acid is transported by retinoic acid binding protein 2 (Crabp2) from the cytoplasm to the nucleus to activate the nuclear retinoic acid receptor, further involved in stem cell differentiation.^29^ Alternatively, under stress induced by alcohol, the Maob promoter sequence acts as Sp/KLF-binding sites, resulting in upregulation of Maob via GAPDH/KLF11 signaling, leading to H_2_O_2_-induced cell death.^30^ Alcohol consumption reduces antimicrobial peptides such as α-defensin 20, 23, 29, and Dmbt1 secretion of the crypts.^6^ The endogenous or exogenous biochemical agents act as antagonists to upregulated Slc7a8, suppressing the secretion of α-defensin vesicles by Paneth cells.^31^ In villi, proteins associated with lipid metabolism are upregulated, unlike lipids anabolism in the liver under alcohol exposure, villi catabolize lipids by upregulating the expression of Aadac and Hsd17b4.^32^ Alcohol upregulates Casp8, which triggers apoptosis^33^ along with Epb41l3. In villi, Maob and retinoic acid-mediated H_2_O_2_-induced cell death and stem cell differentiation are common molecular events under alcohol stress, just like in the crypts. Upon alcohol consumption, some key proteins are also upregulated, including Prxl2a, H-2 MHC-I, and CYP4B1 (IFNγR1). One of the possible explanations for their upregulation is that alcohol damages the villi cell lining, which is now permeable to the gut microbes and attracts immune cells, specifically neutrophils and macrophages that express IFN-γ and IL-17, known key initiators of inflammatory-immune responses triggered by chronic alcohol treatment.^34^ Thus, the IFNγ receptor is upregulated, and the signaling cascades induced by IFNγ increase the expression of MHC-I.^35^ Moreover, Stat1, actin-binding proteins, such as Wdr1, Lcp1, and Colt1, as well as T-cell GTP-binding proteins, such as Gimap3 and Gimap4 are downregulated in the villi of AF as a consequence of villi structural damage.

In conclusion, our spatial proteomics analysis conducted under alcohol treatment has revealed significant modulation in the proteomic profiles of crypts and villi. We identified 4230 proteins in AF samples and 3900 in PF samples, with >3000 proteins present in both crypts and villi under both conditions. Alcohol consumption distinctly modulates proteins and functions of these cellular compartments. Specifically, under alcohol consumption, Paneth cell antimicrobial peptide expression was downregulated, while alcohol metabolism-associated proteins were upregulated. Notably, the upregulated proteins in crypts play a significant role in cellular metabolism and biosynthesis of BHB. In the villi, the upregulation of proteins is related to lipid metabolism and apoptosis alongside the downregulation of actin-filament binding proteins. This study provides valuable insights into the complex interplay between alcohol consumption and proteomic profile changes within the intestinal microenvironment. Further research focusing on the post-translationally modified proteins can further delineate the alcohol-induced biochemical events and their impact on cellular metabolite profiles in crypts and villi.

## Data Availability

The mass spectrometry proteomics data have been deposited to the ProteomeXchange Consortium via the PRIDE^36^ partner repository with the data set identifier PXD048687.

## Supporting Information Available

Effects of alcohol on the integrity of intestinal tissues, number of identified proteins from each group (AF and PF, equivalent to 3000 cells); functions of commonly identified proteins in AF and PF; protein–protein interaction network of uniquely expressed proteins in AF and PF samples; top five biological processes from functional enrichment analysis of the uniquely expressed proteins in AF and PF samples; and number of identified proteins in crypts and villi of AF and PF mice (PDF)
Identified proteins in crypts and villi of AF and PF mice (XLSX)
Statistically significantly changed proteins in crypts and villi between AF and PF mice (XLSX)
