# Supplementary material for: Spatial Proteomics Reveals Alcohol-Induced Damages to the Crypts and Villi of the Mouse Small Intestine
Source: J Proteome Res. 2024 Apr 24;23(5):1801–9. doi: 10.1021/acs.jproteome.4c00037 (PMC11077582; doi:10.1021/acs.jproteome.4c00037)
Supplement: Supplementary file 1 — pr4c00037_si_001.pdf [file pr4c00037_si_001.pdf]

# Spatial Proteomics Reveals Alcohol-Induced Damages to the Crypts and Villi of the Mouse Small Intestine

Patil Shivprasad Suresh,[1] Xinguo Sun,[1] Zhanxiang Zhou,[1,3] Qibin Zhang[1,2]*

[1]Center for Translational Biomedical Research, University of North Carolina at Greensboro, North Carolina Research Campus, Kannapolis, NC 28081, USA.
[2]Department of Chemistry & Biochemistry, University of North Carolina at Greensboro, Greensboro, NC 27402, USA.
[3]Department of Nutrition, University of North Carolina at Greensboro, Greensboro, North Carolina 27402, USA.

**Corresponding Author**

Email: q_zhang2@uncg.edu

**Table of Contents**


| Item | Title | Page Number |
|---|---|---|
| **Figure S1** | (a) The effects of alcohol on the integrity of intestinal tissues. (b) Number of identified proteins from each group (AF and PF, equivalent to 3000 cells). | **S2** |
| **Figure S2** | The functions of commonly identified proteins in AF and PF. | **S3** |
| **Figure S3** | Protein-protein interaction network of uniquely expressed proteins in AF and PF samples. Top five biological processes from functional enrichment analysis of the uniquely expressed proteins in AF and PF samples. | **S4** |
| **Table S1** | Number of identified proteins in crypts and villi of AF and PF mice. | **S5** |
| **Table S2** | Identified proteins in crypts and villi of AF and PF mice. | **XLSX** |
| **Table S3** | Statistically significantly changed proteins in crypts and villi between AF and PF mice. | **XLSX** |

**Supplementary Figure S1.** (**a**) The effects of alcohol on the integrity of intestinal tissues. Villi regions are damaged in alcohol-fed (alcohol-treated) mice. (**b**) Number of identified proteins from each group (AF and PF, equivalent to 3000 cells). DIA-NN was used to process the DIA datasets.

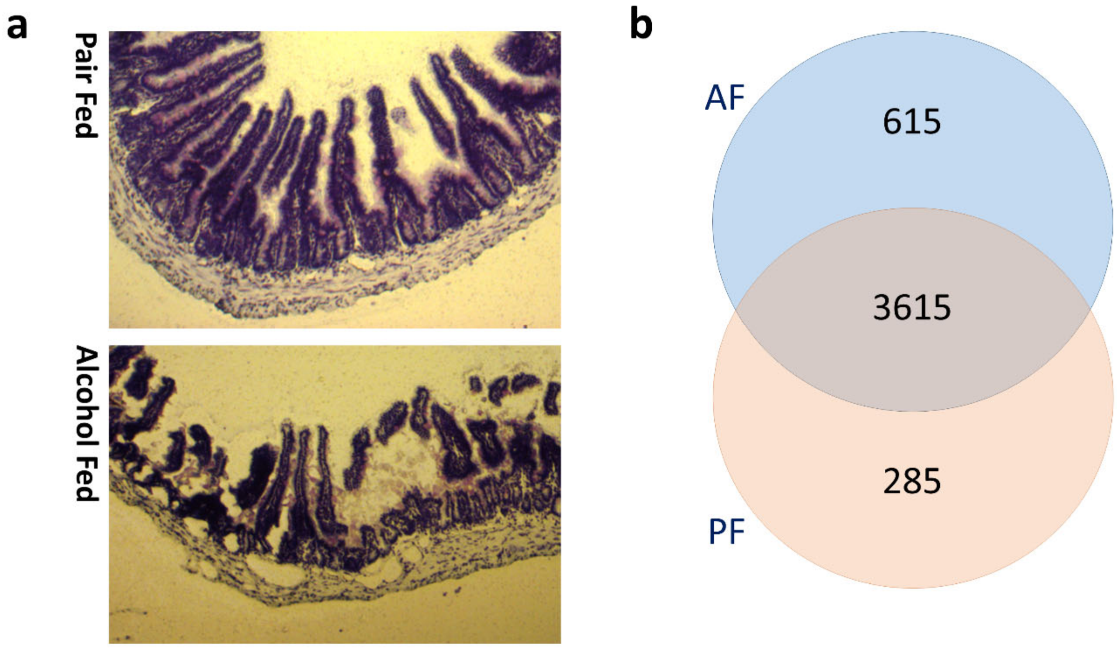

**Supplementary Figure S2.** The functions of commonly identified proteins in AF and PF.

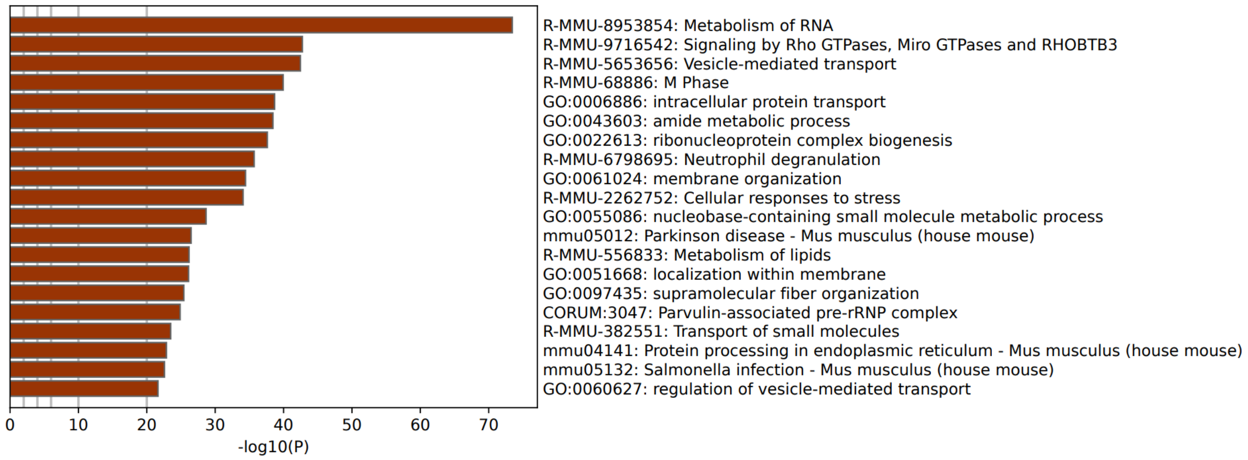

**Supplementary Figure S3.** Protein-protein interaction network of uniquely expressed proteins in AF and PF samples. Top five biological processes from functional enrichment analysis of the uniquely expressed proteins in AF and PF samples.

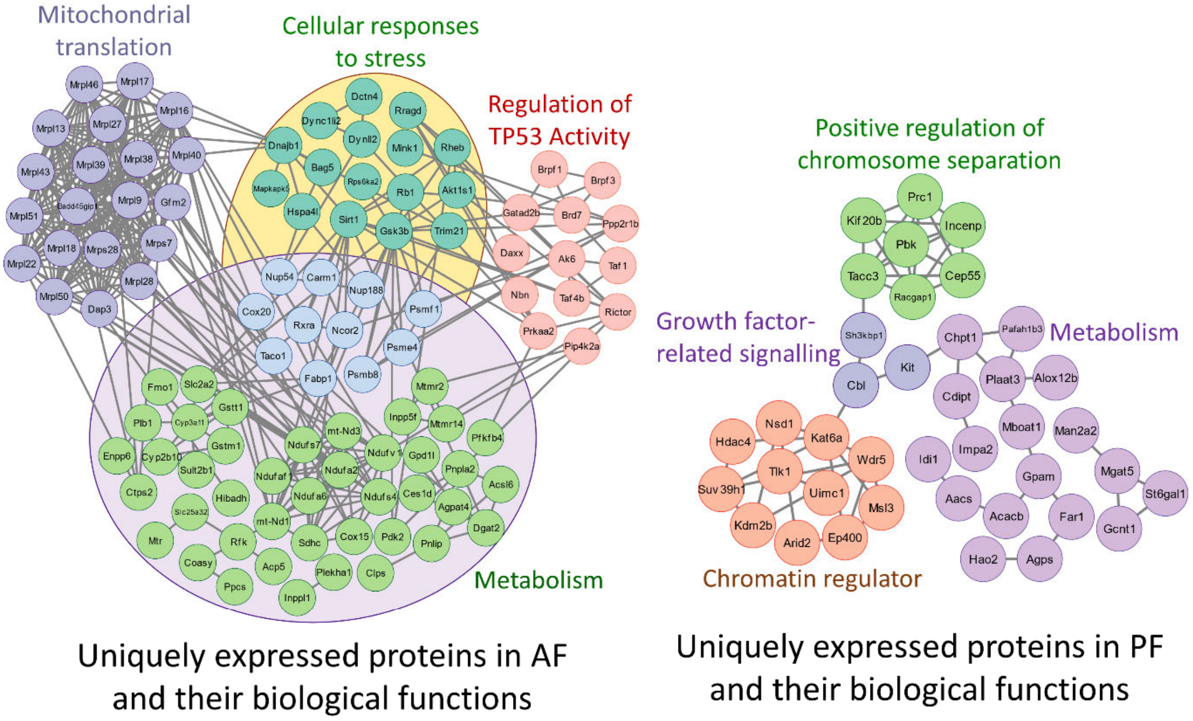


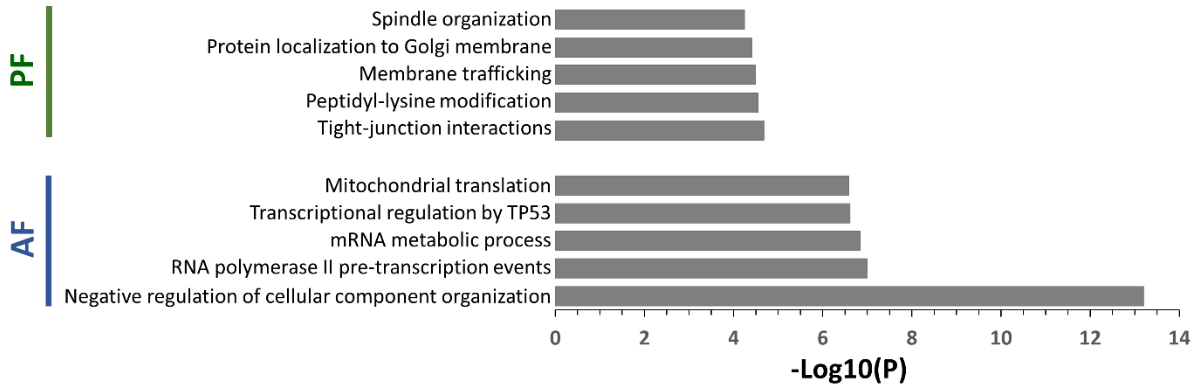

**Supplementary Table S1:** Number of identified proteins in crypts and villi of AF and PF mice.

| Mouse | **AF** (Treatment) | | Mouse | **PF** (Control) | |
|---|---|---|---|---|---|
| | **Crypts** | **Villi** | | **Crypts** | **Villi** |
| | No. of proteins | No. of proteins | | No. of proteins | No. of proteins |
| **1** | 3622 | 3957 | **1** | 3875 | 3381 |
| **2** | 2644 | 4031 | **2** | 3498 | 3644 |
| **3** | 3152 | 3738 | **3** | 3798 | 3758 |
| **4** | 3065 | 3833 | **4** | 2993 | 2999 |
| **5** | 3240 | 3420 | **5** | 3636 | 3448 |
| **6** | 2530 | 3381 | **6** | 2801 | 3863 |
| **7** | 3527 | 3293 | **7** | 3021 | 3688 |
| **8** | 4070 | 3902 | | | |
| **9** | 4085 | 3709 | | | |
